# Prevalence and influencing factors of wheeze and asthma among preschool children in Urumqi city: a cross-sectional survey

**DOI:** 10.1038/s41598-023-29121-x

**Published:** 2023-02-08

**Authors:** Tingting Wang, Haonan Shi, Guangsheng Wan, Zhuohui Zhao, Dan Norback, Guiping Pu, Shaowei Ma, Huijuan Dong, Jian Yao, Junwen Lu, Yue Wang, Qi Yan, Huizhen Qi, Qi Ma, Yufeng Shi

**Affiliations:** 1 School of Nursing & Health Management, Shanghai University of Medicine & Health Sciences, No. 279, Zhouzhu Highway, Pudong New District, Shanghai, 201318 China; 2 School of Public Health, Xinjiang Medical University, Urumqi, 830000 China; 3 Department of Environmental Health, School of Public Health, Fudan University, Shanghai, 200433 China; 4 Key Lab of Public Health Safety of the Ministry of Education, NHC Key Lab of Health Technology Assessment (Fudan University), Shanghai, 200433 China; 5 Department of Medical Sciences, Occupational and Environmental Medicine, Uppsala University, 751 Uppsala, Sweden; 6 Department of Neurology, The Fifth Affiliated Hospital of Xinjiang Medical University, Urumqi, 830000 China; 7 Xinjiang Key Laboratory of Metabolic Disease, Clinical Medical Research Institute, the First Affiliated Hospital of Xinjiang Medical University, Urumqi, 830000 China

**Keywords:** Health care, Risk factors

## Abstract

To investigate the prevalence and indoor environmental influencing factors of wheeze and asthma among preschool children in Urumqi, Xinjiang, China to provide a strong basis for prevention and control. In August 2019, a cross-sectional study involving 8153 preschool children was conducted in 60 kindergartens in Urumqi. The ALLHOME-2 questionnaire was used for childhood wheeze and asthma survey, and the dampness in buildings and health (DBH) questionnaire was used for the childhood home dwelling and living environment survey. Multivariate unconditional logistic regression was then used to analyze the potential influencing factors of childhood asthma and wheeze. The prevalence of wheeze and asthma in children was 4.7% and 2.0%, respectively. Multivariate unconditional logistic regression results suggested that ethnicity other than the Han Chinese (odds ratio (*OR*) 1.39, 95% confidence interval (*CI*) 1.05–1.84), caesarean section (*OR* 1.24, 95% *CI* 1.00–1.53), family history of asthma (*OR* 5.00, 95% *CI* 3.36–7.44), carpet or floor bedding at home (*OR* 1.40, 95% *CI* 1.05–1.87), purchasing new furniture in the mother’s residence during pregnancy (*OR* 1.58, 95% *CI* 1.06–2.36), pet keeping in the residence at aged 0–1 year (*OR* 1.55, 95% *CI* 1.13–2.13), passive smoking by child in the current residence (*OR* 1.35, 95% *CI* 1.01–1.80), and having mould or hygroma in the child's residence at aged 0–1 year (*OR* 1.72, 95% *CI* 1.12–2.64) were risk factors for wheeze. In addition, Girls (*OR* 0.73, 95% *CI* 0.59–0.90) was a protective factor for wheeze. Caesarean section (*OR* 1.46, 95% *CI* 1.06–2.00), family history of asthma (*OR* 7.06, 95% *CI* 4.33–11.53), carpet or floor bedding at home (*OR* 2.20, 95% *CI* 1.50–3.23), and pet keeping in the residence at aged 0–1 year (*OR* 1.64, 95% *CI* 1.04–1.83) were risk factors for asthma, whereas Girls (*OR* 0.58, 95% *CI* 0.42–0.80) was a protective factor for asthma. This survey indicates that the purchase of new furniture, the placement of carpet or floor bedding in the child's residence, the pets keeping, room dampness or moldy phenomena, and passive smoking may all contribute to an elevated risk of wheeze or asthma in children.

## Introduction

Bronchial asthma, referred to as asthma, is a respiratory disease resulting from a variety of causes that often manifests with symptoms, such as wheeze, shortness of breath, chest tightness, and cough^1^. According to a survey of childhood asthma conducted by Asher et al. in 27 centers in 14 countries worldwide, the prevalence of childhood asthma is still on the rise in developing countries, and poses a substantial disease burden^2,3^. In recent years, China has experienced rapid economic development. Furthermore, the tremendous changes that have occurred in people's living environments and ways, resulting in the prevalence of asthma, have increased each year. Asthma not only causes significant damage to the physical health of children but also imposes a severe economic burden on the families of affected children.

Liu et al.^4^ suggested an increasing trend of childhood asthma prevalence in China by comparing the prevalence of childhood asthma over a 20-year period in 16 cities in China. The prevalence was 0.96% in 1990, 1.66% in 2000, and 2.38% in 2010. Xinjiang is a multiethnic aggregation area with a hot and dry climate, and there are large differences in the lives, dietary habits, and residential environments of all ethnic people. In a survey of children from 10 cities in China during 2010–2012, Zhang et al.^5^ concluded that the prevalence of asthma among children was 1.7–9.8%, with the highest prevalence in Shanghai. However, the prevalence of wheeze among children in Urumqi was the highest among these 10 cities. This result suggests a higher prevalence of respiratory disease among children in Urumqi city. This was also confirmed by Wang et al.^6^, who investigated children in Urumqi in 2011 and reported a prevalence of asthma of 3.6%.

According to related studies, the incidence of asthma is strongly associated with genetics, ethnicity, and the quality of the indoor and outdoor environments^7^. However, in recent years, epidemiological surveys of asthma in children in Urumqi city have been scarce. To understand the latest prevalence and risk factors for childhood asthma in Urumqi, a study covering 8153 preschool children in the Xinshi district, Shayibake district, Tianshan district, Shuimogou district, Toutunhe district, and Midong district of Urumqi was conducted in association with the actual situation in August 2019. The prevalence of childhood asthma and indoor environmental risk factors in Urumqi city were investigated. This study aims to provide a theoretical basis and scientific guidance for the prevention of asthma in children in Urumqi and even nationwide.

## Subjects and methods

### Subjects

A stratified cluster random sampling method was used in August 2019, all children with aged 2–8 years in a total of 60 public kindergartens randomly selected from 8 to 12 kindergartens in each of six districts, Xinshi district, Shayibake district, Tianshan district, Shuimogou district, Toutunhe district, and Midong district, according to the administrative region of Urumqi city. A total of 10,000 questionnaires were distributed, all children were equally likely to be included in the study. Finally 8153 valid questionnaires were returned, resulting in an 81.53% response rate. This study was approved by the ethics committee of Fudan University, and all parents of the investigated children gave written informed consent.

### Questionnaire

Contact was made with the Education Bureau of Urumqi city and the kindergarten teachers, and professional training was provided to the responsible teachers involved in the investigated classes before the survey. Questionnaires were administered to the parents of the children by the preschool teachers in the kindergartens, and the questionnaires were completed by the parents or other guardians of the children when they were taken home. They were asked to complete it within a week and return it to the responsible teacher in the preschool. Teachers returned the questionnaires to the kindergarten gardener. The gardener sent the questionnaire to the City Education Bureau of Urumqi after collecting all the questionnaires from the kindergartens where the gardener was located.

The contents of this questionnaire are referred to in ALLHOME-2 in the doctoral thesis of Naydenov^8^ and the questionnaire used by Bornehag on the dampness in construction and health (DBH) survey study^9^. Some revisions were made based on the specific situation in China and Urumqi city. The survey included the following six sections: (1) demographic characteristics, including gender, ethnicity, educational level, home address, etc.; (2) feeding status, including whether they were only children, whether they were breastfed, duration of breastfeeding, and age at which the child attended kindergarten; (3) child and family member health, including wheeze previously, asthma, pneumonia, allergic rhinitis, and other related symptoms; (4) child's residential environment, including housing type, whether there was a renovation, whether furniture was newly purchased, ventilation, smoke evacuation, etc.; (5) lifestyle habits, including animals, plants, cleaning frequency, smoking in the residence of the child, etc.; and (6) dietary habits, including the type of eating, the number of times, etc. Questionnaires were completed by all parents of children, and all questionnaires, excluding ineligible ones, were reviewed by more than two trained subject team members. Excluding questionnaires with less than 80% completion rate and those who did not sign informed consent, 8153 children were finally included in this study.

### Relevant result judgment

In this study, the related outcomes were judged as follows: (1) wheeze: on the basis of the child's spontaneous report of difficulty breathing, objective judgment was performed: the respiratory muscles of the child are all involved in breathing, and the respiratory rate of the child is faster than normal^10,11^; (2) asthma: parental report of a doctor to include ever having a doctor's definite diagnosis of asthma; (3) frequency of the child’s room cleaning where regular cleaning refers to cleaning at least 2 times in 1 week, occasional cleaning refers to cleaning fewer than 2 times in 1 week and more than 1 time in 2 weeks, rare cleaning refers to fewer than 1 cleaning in 2 weeks^7^. (4) passive smoking: weekly exposure of nonsmokers for at least 1 d to smoke emitted from ignited coils or smoke exhaled by smokers^12^. (5) prepregnancy refers to one year before the mother became pregnant.

### Data analysis

Epi Data 3.1 software was used to create the database. One-way *χ*^*2*^ tests were performed using SPSS 25.0 software. Multivariate logistic regression models were used to analyse potential respiratory disease risk factors. The confounding factors included in this study were those factors that can be found statistically significant in the univariate model as shown in Tables 3 and 4. Differences with *P* < 0.05 were considered statistically significant.

### Ethics approval and consent to participate

The study was conducted strictly in accordance with the Declaration of Helsinki and approved by the research ethics committee of Fudan University (protocol no. IRB00002408 & FWA00002399), all parents and class teachers of the children under investigation have signed written informed consent.

## Results

### General condition of the investigated subjects

A total of 8153 preschool children were investigated in this study, including 4235 boys (51.9%) and 3918 girls (48.1%). Among them, the youngest age was 2.00 years, and the oldest age was 7.83 years, with a mean age of 5.27 ± 1.10 years (Table 1). Additionally, 7081 (86.9%) were Han Chinese, and 1072 (13.1%) were members of other ethnic groups.

**Table 1:** Comparison of wheeze and asthma among preschool children with different characteristics in Urumqi (*n* = 8153).

Characteristics	Number of individuals	Number of wheeze cases	Prevalence (%)	*χ*^2^	*P*	Number of asthma cases	Prevalence (%)	*χ*^2^	*P*
Gender									
Boys	4235	227	5.4	8.30	< 0.01	107	2.5	10.05	< 0.01
Girls	3918	157	4.0			60	1.5		
Ethnicity									
Han Chinese	7081	312	4.4	11.07	< 0.01	131	1.9	10.56	< 0.01
Other	1072	72	6.7			36	3.4		
Age (years)									
2 ~ 4	1033	46	4.5	0.22	0.90	23	2.2	0.37	0.83
4 ~ 6	4828	231	4.8			100	2.1		
6 ~ 8	2292	107	4.7			44	1.9		
Type of birth delivery									
Normal childbirth	4187	178	4.3	4.04	0.05	73	1.7	3.99	0.05
Cesarean delivery	3966	206	5.2			94	2.4		
Only child									
Yes	3584	169	4.7	0.00	0.98	69	1.9	0.48	0.49
No	4569	215	4.7			98	2.1		
Total	8153	384	4.7			167	2.0		

### Wheeze and asthma prevalence

Approximately 4.7% of children reported symptoms of previous wheeze, and the prevalence of physician-diagnosed asthma was 2.0% (Table 1). After adjustment for age, the prevalence was 4.6% for wheeze and 2.1% for asthma. Among them, boys had higher rates of wheeze and asthma than girls (*P* < 0.01), other ethnic groups had higher rates of wheeze and asthma than Han Chinese (*P* < 0.01), and children born via caesarean section had higher rates of wheeze and asthma than normal childbirth children (*P* < 0.01).

### Univariate analysis of wheeze and asthma with indoor environmental variables

The analyses revealed that carpet or floor bedding at home, new furniture acquisition in the mother's prepregnancy residence, mould or damp phenomenon in the mother's prepregnancy residence, new furniture acquisition in the mother's prepregnancy residence, new furniture acquisition in the child's residence at aged 0–1 year, grooming or dampness in the residence at aged 0–1 year, pet keeping in the residence at aged 0–1 year, passive smoking by child in the current residence, passive smoking by child at aged 0–1 year in the residence, and passive smoking by mothers in their residence during pregnancy were the main environmental factors influencing the prevalence of children's wheeze (*P* < 0.05).

Carpets or floor bedding at home, grooming in the mother's prepregnancy residence, mouldy in the mother's prepregnancy residence, dampness in the mother's prepregnancy residence, addition of new furniture before pregnancy, home renovation before pregnancy, new furniture in the home at aged 0–1 year, grooming in the child's prepregnancy residence at aged 0–1 year, mouldy in the residence at aged 0–1 year, dampness in the residence at aged 0–1 year, pet keeping in the residence at aged 0–1 year, and passive smoking by mothers in their residence during pregnancy were the main environmental factors influencing the prevalence of childhood asthma (*P* < 0.05). They are all shown in Table 2.

**Table 2:** Univariate analysis of wheeze and asthma with indoor environmental variables (*n* = 8153).

Factors	Wheeze				Asthma			
	Number of cases	%	*χ*^2^	*P*	Number of cases	%	*χ*^2^	*P*
Residence area								
< 75m^2^	99	5.0	0.47	0.50	43	2.2	0.19	0.66
≥ 75m^2^	285	4.6			124	2.0		
Carpet or floor bedding at home								
Yes	66	6.9	11.37	< 0.01	42	4.4	29.36	< 0.01
No	318	4.4			125	1.7		
Addition of new furniture before pregnancy								
Yes	108	6.1	9.19	< 0.01	43	2.4	1.49	0.22
No	276	4.3			124	1.9		
Home renovation before pregnancy								
Yes	83	6.9	15.27	< 0.01	37	3.1	7.51	< 0.01
No	301	4.3			130	1.9		
Mouldy or dampness before pregnancy								
Yes	63	7.2	13.47	< 0.01	36	4.1	20.78	< 0.01
No	321	4.4			131	1.8		
Addition of new furniture during pregnancy								
Yes	72	8.3	27.37	< 0.01	31	3.6	11.05	< 0.01
No	312	4.3			136	1.9		
Home renovation during pregnancy								
Yes	51	8.7	22.18	< 0.01	21	3.6	7.33	< 0.01
No	333	4.4			146	1.9		
Mouldy or dampness during pregnancy								
Yes	54	7.9	17.07	< 0.01	29	4.3	18.02	< 0.01
No	330	4.4			138	1.8		
Addition of new furniture at child’s aged 0–1 year								
Yes	52	7.1	10.47	< 0.01	28	3.8	12.82	< 0.01
No	332	4.5			139	1.9		
Home renovation at child’s aged 0–1 year								
Yes	35	7.2	7.15	< 0.01	23	4.7	18.56	< 0.01
No	349	4.6			144	1.9		
Mouldy or dampness at child’s aged 0–1 year								
Yes	56	9.2	29.07	< 0.01	26	4.2	15.92	< 0.01
No	328	4.3			141	1.9		
Use of air conditioning								
Yes	115	4.5	0.36	0.55	50	2.0	0.16	0.69
No	269	4.8			117	2.1		
Pet keeping in the child’s residence								
Yes	63	4.6	1.62	0.20	32	2.8	3.46	0.06
No	321	5.4			135	1.9		
Flowering plants grown in the child's residence								
Yes	115	4.7	0.01	0.93	50	2.0	0.00	0.96
No	269	4.7			117	2.1		
Pet keeping in the residence at child’s aged 0–1 year								
Yes	50	7.6	13.14	< 0.01	24	3.6	9.03	< 0.01
No	334	4.5			143	1.9		
Flowering plants grown in the residence at child’s aged 0–1 year								
Yes	97	5.2	1.27	0.26	35	1.9	0.36	0.55
No	287	4.6			132	2.1		
Passive smoking by child in current residence								
Yes	179	6.0	18.10	< 0.01	72	2.4	3.30	0.07
No	205	4.0			95	1.8		
Passive smoking by child at aged 0–1 year in residence								
Yes	150	6.1	15.03	< 0.01	56	2.3	0.90	0.34
No	234	4.1			111	2.0		
Passive smoking during pregnancy								
Yes	128	6.2	13.98	< 0.01	55	2.7	5.35	0.02
No	256	4.2			112	1.8		
Room cleaning frequency								
Often	316	4.5	4.07	0.13	135	1.9	5.41	0.07
Occasionally	63	5.9			28	0.3		
Very seldom	5	6.1			4	0.0		
Family history of asthma								
Yes	36	9.4	106.83	< 0.01	23	13.7	115.80	< 0.01
No	348	4.4			144	1.8		

### Multivariate analysis of wheeze and indoor environmental variables

Wheeze illness was used as the dependent variable (0 = no; 1 = yes), variables significant in univariate analysis were introduced into the logistic regression model, and 9 variables from the final entry main effect model were associated with the incidence of wheeze. Among these, ethnicity other than the Han Chinese, caesarean section, family history of asthma, carpet or floor bedding at home, acquisition of new furniture in the mother's residence during pregnancy, pet keeping in the child's residence at aged 0–1 year, and passive smoking by child at aged 0–1 year in residence were risk factors for wheeze. Girls was a protective factor for wheeze (Table 3).

**Table 3:** Multivariate logistic regression analysis of risk factors for wheeze among preschool children in Urumqi (*n* = 8153).

Factors	Reference	*P*	*OR*	95% *CI*
Gender	Boys	< 0.01	0.73	0.59 ~ 0.90
Ethnicity	Han ethnic group	0.02	1.39	1.05 ~ 1.84
Type of birth delivery	Normal childbirth	0.05	1.24	1.00 ~ 1.53
Family history of asthma	No	< 0.01	5.00	3.36 ~ 7.44
Carpet or floor bedding at home	No	0.02	1.40	1.05 ~ 1.87
Addition of new furniture during pregnancy	No	0.02	1.58	1.06 ~ 2.36
Pet keeping at child’s aged 0–1 year in the residence	No	< 0.01	1.55	1.13 ~ 2.13
Passive smoking by child in current residence	No	0.04	1.35	1.01 ~ 1.80
Mouldy or dampness at child’s aged 0–1 year	No	0.01	1.72	1.12 ~ 2.64

*OR* odds ratio, *CI* confidence interval.

### Multivariate analysis of asthma and indoor environmental variables

The presence or absence of asthma was used as the dependent variable (0 = no; 1 = yes), variables significant in univariate analysis were introduced together into the logistic regression model, and 5 variables from the final entry main effect model were associated with the incidence of asthma. Among these, caesarean section, family history of asthma, carpet or floor bedding at home, and child's aged 0–1 year were risk factors for asthma when keeping a pet in the residence. Girls was a protective factor for asthma (Table 4).

**Table 4:** Multivariate logistic regression analysis of risk factors for asthma among preschool children in Urumqi (*n* = 8153).

Factors	Reference	*P*	*OR*	95%*CI*
Gender	Boys	< 0.01	0.58	0.42 ~ 0.80
Type of birth delivery	Normal childbirth	0.02	1.46	1.06 ~ 2.00
Family history of asthma	No	< 0.01	7.06	4.33 ~ 11.53
Carpet or floor bedding at home	No	< 0.01	2.20	1.50 ~ 3.23
Pet keeping in the residence at child’s aged 0–1 year	No	0.03	1.64	1.04 ~ 1.83

*OR* odds ratio, *CI* confidence interval.

## Discussion

The results of this survey showed that the prevalence of wheeze (4.6%) and asthma (2.1%) among preschool children after adjustment for age in Urumqi city is high. In addition to genetic factors, wheeze and the onset of asthma have extremely strong associations with indoor environmental factors.

Kim et al.^13^ found a higher prevalence of asthma in boys than in girls, and children with a family history of asthma had a higher risk of developing asthma. Quite a few studies have found that the risk of asthma in minorities is greater than that in the Han population^7,14^. This is consistent with the results of the present study. Genetic factors are one of the important contributors to the pathogenesis of asthma, and more than 100 candidate genes for asthma have been identified thus far. Among them, genes, such as ADRB2, interleukin 4, IL-13, and ormdl3, in the chromosome 5q region are more clearly associated with childhood asthma onset^15^. These factors may have contributed to the fact that gender, ethnicity, and family history of asthma are risk factors for the development of childhood asthma. In addition to genetic factors, differences in lifestyle habits and residential environments among different ethnic groups and genders may also contribute to the predisposition of girls and ethnic minorities to asthma. However, the clustering of affiliation minorities in Xinjiang, with a higher difference in dietary habits and lifestyle habits than other provinces and municipalities, may be the reason why the preschool children in Urumqi city have different prevalence of asthma and wheeze from other regions. For example, there is a strong desire to lay carpets and floor pads, all of which contribute to the breeding of bacteria and dust mites in the minority families of Xinjiang, which, combined with the fact that they are not easily cleaned, children are usually exposed to a large number of bacteria and dust mites inhaled into their bodies, resulting in an elevated incidence of respiratory diseases^6^. This survey similarly found carpet or floor mats lying at home to be a risk factor for wheeze and asthma. In addition, the differences in life, dietary habits, and residential environments between minorities in Urumqi, Xinjiang, China, and the Han ethnic group may also contribute to the higher prevalence of asthma and wheeze in minorities than in the Han ethnic group. Previous studies found that the prevalence of asthma in children is higher among boys than girls before puberty^16,17^. One reason may be that boys had a greater amount and range of activities, which have more opportunities for allergen exposure^18,19^. Another reason may be related to the differences in hormone secretion between men and women than girls^20^. In addition, boy's physiology or respiratory tract which are shown to have smaller diameter than girls^21^. It was also found in the multivariate analysis of this study. Additionally, type of birth delivery was found to be a risk factor for wheeze and asthma prevalence in this study. Studies have confirmed that type of birth delivery has a very important impact on the development and maturation of the neonatal immune system^22^. Caesarean delivery of newborns increases the risk of allergic diseases later in infancy due to a lack of exposure to the normal flora of the maternal vagina and gut; instead Escherichia coli and Fusobacterium enter the neonatal gastrointestinal tract at an early age^23,24^. Therefore, we considered that the prevalence of wheeze and asthma was higher in children who were delivered by caesarean section than in those who were delivered vaginally, and the reasons may be related to this.

Due to rapid economic development in recent years, people also have an increasing demand for indoor renovation and furniture. However, whether decorating materials or newly purchased furniture, which contains substances, such as formaldehyde, benzene, toluene, and xylene, children inhale such substances in excess and irritate their airways, increasing the risk of developing asthma^25^. Although indoor renovation and newly purchased furniture were not found to be associated with asthma incidence in children in this study, newly purchased furniture in mothers' gestational residence was found to result in an elevated risk of developing wheeze. This may be due to the ability of formaldehyde to transport across the placenta endangering the health of the foetus, and pregnant women have been in an environment containing substances, such as formaldehyde, for a long time, which can cause abnormal foetal development and even foetal malformations^26^. Indoor passive smoking has been confirmed to be closely associated with child health in many studies. A cohort study by Robison et al.^27^ in Boston showed that passive smoking may affect foetal and infant lung development, resulting in impaired lung function from early in life and increasing the number of wheeze episodes in infants and young children. A meta-analysis of 79 cohort studies by Burke et al.^28^ also suggested passive smoking as a risk factor for the development of wheeze in children. This conclusion was similarly reached in the present investigation.

Domestic pets, which are now very common in our country, but many studies at home and abroad have confirmed that pets increase the levels of endotoxin to allergens in the environment and induce asthma after inhalation in children^29,30^. The present study also identified pet keeping in the residence when the child was 0–1 years old as a risk factor for wheeze and the development of asthma. It is worth mentioning that there are still quite a few studies proposing that exposure to endotoxin early in life is a protective factor for asthma pathogenesis^31,32^. The reason for this difference, we consider may be due to the dry climate in Xinjiang, which facilitates for the spread of allergens via keeping pets. Therefore, keeping pets at home can lead to an elevated risk of wheeze and asthma.

However, this survey also found that a wet environment was similarly a risk factor for the incidence of wheeze. A survey by Zhao et al.^33^ in Taiyuan, Shanxi, showed that signs of dampness or indoor mould was positively associated with asthma and allergic disease in nearly all children. This may be related to the fact that the humid environment is prone to bacterial and dust mite breeding, so overly dry or humid shelter should be avoided.

There are certain limitations in this study. Since the present study is a cross-sectional survey study, causal arguments are less able. In addition, we only considered the influence of indoor environmental risk factors on asthma and wheeze in preschool children, and did not take into account outdoor environmental factors and the exposure of children to them in preschool. Children with asthma were not excluded from the analyses with regard to children with wheeze. In addition, childhood asthma is influenced by both genetic and environmental factors, but we focused on the association between indoor environmental factors and asthma or wheeze and did not explore genetic and environmental factors too much. Finally, since there were some children in this study who did not sign the informed consent form or had incomplete responses to the questionnaire, it may lead to selection bias in prevalence and results of multivariate analysis. Therefore, it is necessary to investigate more influencing factors through methods such as case–control study, genome sequencing and so on in subsequent studies.

In summary, the focus should be on children with a family history of asthma, and parents should reduce the purchase of new furniture before conception, during pregnancy, and at aged 0–1 year for their child. Avoiding carpet or floor bedding in the child's residence, keeping pets, actively spreading out dewetting work on the room and avoiding passive smoking by mothers and children to start with an active indoor environment might be able to reduce the risk of wheeze and asthma in children.

## Conclusion

In addition to individual factors and family history of asthma, there is a strong association between indoor environmental factors and the prevalence of wheeze and asthma in preschool children. Further expansion of longitudinal studies and cohort studies could help to validate and gain insight into the influence of indoor environmental factors on wheeze and asthma in children.

## Data Availability

The datasets generated and/or analysed during the current study are not publicly available due the data belongs to the School of Public Health of Fudan University but are available from the corresponding author on reasonable request.

## Abbreviations

*CI*: Confidence interval
*OR*: Odds ratio
